# Does the organizational model of the maternity health clinic have an influence on women’s and their partners’ experiences? A service evaluation survey in Southwest Finland

**DOI:** 10.1186/1471-2393-12-96

**Published:** 2012-09-14

**Authors:** Miia Tuominen, Anne Kaljonen, Pia Ahonen, Päivi Rautava

**Affiliations:** 1 Public Health Department, University of Turku, FI-20014 Turun yliopisto, Turku, Finland; 2 Turku Institute for Child and Youth Research, University of Turku, FI-20014 Turun yliopisto, Turku, Finland; 3 Health Care Faculty, Turku University of Applied Sciences, Ruiskatu 8, 20760, Turku, Finland; 4 Turku Clinical Research Centre, Turku University Hospital, PO Box 52, 20521, Turku, Finland

**Keywords:** Health services research, Primary health care, Comparative study, Pregnancy, Maternal health services, Prenatal care, Continuity of patient care, Parents

## Abstract

**Background:**

In high-income countries, great disparities exist in the organizational characteristics of maternity health services. In Finland, primary maternity care is provided at communal maternity health clinics (MHC). At these MHCs there are public health nurses and general practitioners providing care. The structure of services in MHCs varies largely. MHCs are maintained independently or merged with other primary health care sectors. A widely used organizational model of services is a combined maternity and child health clinic (MHC & CHC) where the same public health nurse takes care of the family from pregnancy until the child is at school age. The aim of this study was to determine how organizational model, MHC independent or combined MHC & CHC, influence on women’s and their partners’ service experiences.

**Methods:**

A comparative, cross-sectional service evaluation survey was used. Women (N = 995) and their partners (N = 789) were recruited from the MHCs in the area of Turku University Hospital. Four months postpartum, the participants were asked to evaluate the content and amount of the MHC services via a postal questionnaire. Comparisons were made between the clients of the separate MHCs and the MHCs combined to the child health clinics.

**Results:**

Women who had used the combined MHC & CHCs generally evaluated services more positively than women who had used the separate MHCs. MHC’s model was related to several aspects of the service which were evaluated “good” (the content of the service) or “much” (the amount of the service). Significant differences accumulated favoring the combined MHC & CHCs’ model. Twelve aspects of the service were ranked more often as “good” or “much” by the parents who had used the combined MHC & CHC, only group activities regarding delivery were evaluated better by women who had used the separate MHCs.

**Conclusions:**

Based on the women’s and partners’ experiences an organizational model of the combined MHC & CHC where the same nurse will take care of family during pregnancy and after birth of the child was preferred. This model also provides greater amount of home visits and peer support than the separate MHC.

## Background

The organizational framework of maternity care services varies greatly in European countries. Despite this disparity, many positive aspects - in terms of maternal and infant health - could be reached [1]. This observation is confirmed by several studies evaluating the relationship between the organizational features of the maternity care services, such as professional education of the main care provider [2,3], number of visits [4,5] or model of the care [6-8], and pregnancy or infant outcomes. Thus, the one defining feature that could give consistent quality in maternity care services cannot be easily found. In Finland, primary maternity care is provided by public maternity and child health clinics [9] that were mandated by law in 1944 to guarantee free health care services for every pregnant woman and all children under school age. From the onset, maternity health clinics (MHCs) provided community-based ante- and postnatal care and were led by midwives and physicians. From 1972, due to the Public Health Act, MHC services were carried out as part of the newly established municipal health center and were usually led by the public health nurses (PHNs) with general practitioners (GPs).

Finnish PHNs are registered nurses who are specialized in public health nursing. The health promotion and prevention of illnesses during the lifespan of the individuals and throughout the communities are the core tasks of PHNs’ work. The education of PHN takes four years (Bachelor’s Degree, 240 ECTS) and it encompasses all sectors of the clinical competence of the public health nursing: family planning, maternity and child health care, school and occupational health care, and home nursing [10]. The responsibilities of PHN are independent in Finnish primary health care. In the MHCs and child health clinics (CHCs), PHNs are practicing as the main care providers. GPs have their own role as the medical experts, providing health promotion and care for the women, children, and the whole families during scheduled and additional check-ups. Good collaboration and consulting between PHNs and GPs are crucial. In addition, midwives are able to work in MHCs in Finland and, in that case, they are ordinarily also qualified as PHN’s. At present, the majority of the MHC’s nurses have a PHN’s degree (75%). Both PHN’s and midwives degree have a fifth (20%) of the nurses. The number of the MHC’s nurses who have only the midwife’s degree is quite small (5%) [11].

In Finland, a woman’s first antenatal visit to a MHC takes place on average in the tenth week of gestation. The services of a MHC are frequently used by the families. In the year 2010, the mean of all antenatal visits was 15.6. There is a notable variation in the total amount of the antenatal visits among different hospital districts in Finland [12].

The care for families at MHCs is highly variable. Present law regarding the work in a MHC does not define the manner in which the work should be organized and hence there is great variation in MHC services in Finland, even within the same health care center. MHCs are organized mainly in three ways: as separate MHCs focusing solely on maternity care, as combined to the family planning clinic services, or as combined to the CHCs where the same PHN will take care of the family from the pregnancy until the child is at school age. The GP could be the same at MHC and CHC but in practice there is a large turnover with GPs in primary health care. GPs do not typically follow the same family as would a PHN. Thus, the continuity of care in the MHC and CHC services is mainly implemented by PHNs. According to a recent national survey, in 16% of the Finnish municipalities, the MHC services were organized into separate clinics, in 33% as combined clinics with family planning services, and in 20% as combined into the CHCs. Other methods to organize MHC services were implemented in 31% of the municipalities [11]. The relationship between the number of the antenatal visits and the organizational model of the MHC is largely unknown.

In Finland, there is discussion about which organizational models are the most effective for MHCs. Experts have not agreed whether maternity care services should be developed as separate clinics focusing on women’s reproductive health issues [13,14] or combined with children’s and families’ health and welfare services [15,16]. Lack of evidence showing the benefits and weaknesses of different MHC models make the consensual development of the MHC services challenging.

When developing maternity care services, the scope should be focused on health and economic outcomes as well as on interventions, the processes, and the context of the care [17-19] and on the care-receivers’ experiences [20,21]. The user’s satisfaction with maternity services has been widely investigated, and there is evidence that European women [22-24] and men [25] are mainly satisfied with the primary maternity care services. Previously Finnish studies related to MHC services found variation in families’ experiences. Viljamaa [26] discovered that parents were satisfied with the services of the MHC and CHC, especially with the manner of PHNs’ actions and the confidential atmosphere of the clinics. Similarly, in a recent study of the National Institute for Health and Welfare [27], the majority of parents ranked services of the MHC and CHC as good or excellent. Former findings regarding MHC services ranged from women’s very good experiences [28] and comprehensive sense of control [29] to parents’ ambiguous descriptions of dissatisfaction such as mistreatment by the staff and lack of individualized attention and information [30,31].

Although the combined MHC & CHCs have been operating in Finland over forty years, little is known about how the connecting of maternity care to child health care effects the parents’ experiences or the health outcomes of the mother and the baby. The aim of this study was to compare two models of the primary maternity health care services: the separate MHCs and the combined MHC & CHCs in relation to parents’ experiences. The research question was: does the organizational model of the MHC affect women’s and their partners’ experiences regarding the services of the MHC?

## Methods

### Design and sample

A comparative service evaluation design was used. The study was part of the multidisciplinary STEPS-study which is being carried out in the area of the Turku University Hospital by the Institute for Child and Youth Research at the University of Turku [32]. The Turku Institute for Child and Youth Research focuses on the health and welfare of families. The STEPS-study is based on a cohort of approximately 2000 children and their families from Southwest of Finland that will be followed up until the children are young adults. The women participating in the STEPS-study were recruited in early pregnancy in the MHCs from September 2007 to August 2009, and at the hospital during the intrapartum care from September 2007 to March 2010. Both non-Finnish and non-Swedish speaking persons were excluded (N = 661). The letter of consent was supplied to the partners by the women. The STEPS-study protocol was approved by the Ethical Committee of the Turku University Hospital in June 2007 and by the Ministry of Social Affairs and Health in April 2008. Altogether 1797 (18.3%) women out of all parturients in the area of Turku University Hospital of the same period (N = 9811) participated in the STEPS-study.

The present data were collected from women and their partners four months postpartum using a postal questionnaire. Data regarding women’s own and their partners’ background characteristics and families’ socio-economic situation in the early pregnancy were also used. The study group included altogether 995 women and 789 partners (all men) who participated in the STEPS-study and had their first visit at a maternity health clinic from January 2008 to May 2009. The research period was defined in order to ensure that the antenatal care of all participants took place during the years 2008 and 2009.

**Figure 1:**
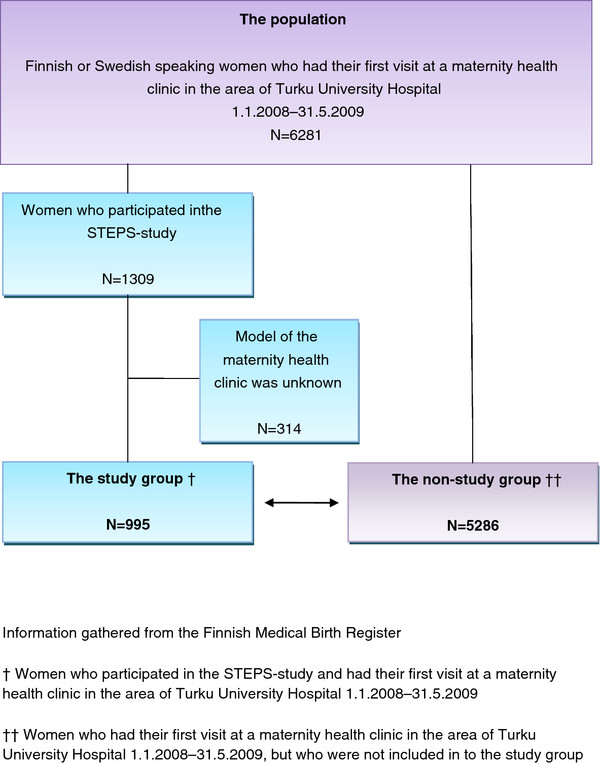
The formation of the study group.

In the dropout analysis, the background characteristics of the study participants (women) were compared with the data of non-participants who had their first visit at a maternity health clinic in the area of Turku University Hospital during the same period. Their background data were gathered from the Finnish Medical Birth Register. The formation of the study group is described in the Figure 1.

Comparison settings were built between the two models of the MHC services: separate MHCs and combined MHC & CHCs. The outcome measures of the settings were participants’ experiences regarding the content and the amount of the received MHC services.

### Measures

Background information regarding the organizational models of the MHC services was gathered via a survey to the administrators of health care centers (N = 17) in the spring of 2010. The administrators were asked whether the MHC services were carried out separately or combined with the CHC services. The enquiry covered the years 2008 and 2009. Required information was received from all health care centers covering the MHC units of 28 municipalities. Data from three small municipalities had to be excluded due to the inability to interpret their findings.

**Figure 2:**
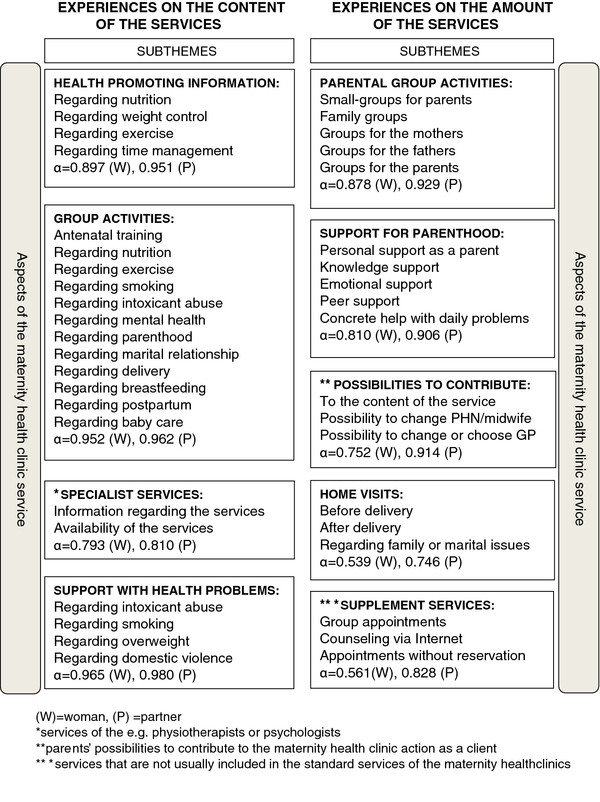
Constitution of the themes of the questionnaire and Cronbach’s α coefficients of the sum variables.

The questionnaires for the women and their partners included previously validated questions from the study of Viljamaa [26] evaluating MHC and CHC services in Central Finland from the point of view of supporting parenthood, family-centered services, and peer groups. Questions regarding different aspects of the MHC service were selected and modified for this study by the expert team of the 10Points-project of the Turku University of Applied Sciences [33]. The questionnaire contained 42 questions that covered the two main themes and nine subthemes focused on the content and amount of the MHC services (Figure 2). The participants were asked to evaluate their experiences of the content of MHC services on a five-point Likert scale ranging from 1 (very poor) to 5 (very good) and experiences of the amount of MHC services received on a five-point Likert scale ranging from 1 (none or very little) to 5 (very much). The theoretical maximum of every scale was 5.

### Analytic strategy

For the statistical analysis, the data were classified according to the model of the service into two groups: separate MHCs and combined MHC & CHCs. The MHC units that had both models in the same clinic were excluded. The determinant was the connection to the child health clinic services. Those MHCs that were connected to other primary health care services, such as family planning clinic or home nursing, were classified into the MHCs.

The data was analyzed statistically using SPSS 18.0 and SAS Release 9.1. for Windows. Based on the subthemes of the questionnaire the sum variables were constituted and the reliability of the measure was evaluated using Cronbach’s α coefficient (Figure 2). Cronbach’s alpha for the whole measure was with women 0.935 and with partners 0.965.

Descriptive statistics were calculated in terms of frequency, percent distribution, and mean and standard deviation. The limit for statistical significance was set at p < 0.05. The T-test for independent samples, Mann-Whitney’s U-test and ANOVA were used to determine if there were any differences between the groups of the participants. In addition, the dichotomy classification of the variables was performed. The outcome variables regarding the experiences of the MHC service were classified as “good” (very good + good) and “poor or neutral” (neither good nor poor + poor + very poor) and regarding amount of the MHC service as “much” (very much + much) and “little” (none or very little + little + neither little nor much). Values “good” and “much” were set to indicate satisfaction with the service. These cut-off points were used to identify the areas for development in the MHC service. The χ^2^ test and Fisher’s exact test were used to compare differences between the groups. Binary logistic regression analysis was used to determine a relation between the participants’ sociodemographic background and the dichotomic dependent variables. The confidence interval was set to 95% in all analyses.

## Results

### Sociodemographic background of the participants

The essential sociodemographic variables of the study group and the non-study group (a cohort of parturients in Southwest Finland) are presented in Table 1. The study group represents accurately the non-study group in relation to the obstetric background variables. However, the participants were a little older, more often nulliparous and married, and more of them were working in a professional occupation than of the non-participants. The majority of the participants used the services of the separate MHCs (N = 740, 76.4%) and nearly a quarter (N = 228, 23.6%) the combined MHC & CHCs.

**Table 1:** Background characteristics of the women

	**Study group** †	**Non-study group** ††	P*
	**n (%)**	**n (%)**	
**Woman (n)**	995	5286	
**Age, mean, years (SD)**	30.8(4.503)	30.0(5.199)	<0.001
	min 17.1-max 44.0	min 14.9-max 49.5	
**Civil status**			
Married	600(60.3)	2848(53.9)	0.001
Unmarried	389(39.1)	2387(45.2)	
Other	6(0.6)	51(1.0)	
**Occupation**			
Professional occupation	165(16.6)	536(10.1)	<0.001
Service occupation	85(8.5)	390(7.4)	
Worker	32(3.2)	170(3.2)	
Other/unknown	713(71.7)	4190(79.3)	
**Number of deliveries**			
0	541(54.4)	2324(44.0)	<0.001
1	306(30.8)	1877(35.5)	
2 or more	148(14.9)	1085(20.5)	
**Abortions**			
Yes	124(12.5)	781(14.8)	0.059
No	869(85.2)	4501(85.2)	
**Gestational age (days) mean (SD)**	278.0(12.966)	278.0(13.526)	0.949
**Delivery**			
Vaginal	769(77.3)	4085(77.3)	0.416
Breech birth	14(1.4)	48(0.9)	
Vacuum or forceps extraction	78(7.84)	458(8.7)	
Section	134(13.5)	695(13.2)	
**Baby’s birth weight (g) mean (SD)**	3486.8(550.9)	3513.1(567.8)	0.179
	min 420-max 5350	min 230-max 6040	

Information gathered from the Finnish Medical Birth Register.

†) Women who participated in the STEPS-study and had their first visit at a maternity health clinic in the area of Turku University Hospital 1.1.2008–31.5.2009.

††) Women who had their first visit at a maternity health clinic in the area of Turku University Hospital 1.1.2008–31.5.2009, but who were not included in the study group.

*) Used statistical test: Pearson’s Chi-Square and T-test for independent samples.

### Parents’ experiences of the maternity health clinic services

The reported total score mean (SD) of the service experiences (both amount and content of the MHC service) was for women 2.42 (0.519) and for partners 2.37 (0.660).

When considering aspects of the service (see Figure 2), the subtheme of the health promoting information was evaluated best 3.21 (0.802) and the supplement services the worst 1.35 (0.594) by the women. The means of the other subthemes were: support for parenthood 3.17 (0.803), support with health problems 3.13 (0.717), group activities 3.03 (0.812), specialist services 2.92 (0.931), possibilities to contribute as a client 1.55 (0.805), home visits 1.54 (0.763), and parental group activities 1.37 (0.714).

From the partners, the subtheme of the group activities received the highest score 3.13 (0.802) and the supplement services the lowest 1.63 (0.839). Means of the other subthemes according to partners were: support with health problems 3.02 (0.757), health promoting information 3.01 (0.791), specialist services 2.94 (0.842), support for parenthood 2.85 (0.883), possibilities to contribute as a client 1.77 (0.960), parental group activities 1.69 (0.888), and home visits 1.68 (0.892).

### The relation between the model of maternity health clinic and the parents’ experiences

For the women who have used the services of the separate MHC, the total score mean (SD) of the service experiences was 2.40 (0.513) and for partners 2.36 (0.649). When considering the combined MHC & CHCs, the women’s mean score was 2.50 (0.537) and partners’ 2.43 (0.698). Women who had used the combined MHC & CHCs were more satisfied with the service than women who had used the separate MHCs [F(1,718) = 4.579, p = 0.033]. There was no significant difference between the partners who have used the separate MHCs or the combined MHC & CHCs [F(1,622) = 1.269, p = 0.260].

The percentage values of the dichotomized variables were examined in relation to the MHC model. Participants evaluated the content of the MHC services mainly “poor or neutral” (= the proportion of the “poor or neutral” evaluations over 50%) regardless of the MHC model. As an exception, the health promoting information regarding nutrition was evaluated more “good” (= the proportion of the “good” evaluations over 50%) by the women regardless of the MHC model.

The majority of the participants assessed the amount of received MHC services as “little” regardless of the model of the MHC. Yet, an exception was the amount of the service aspect “knowledge support” which was evaluated more often as “much” (= the proportion of the “much” evaluations over 50%) by the women regardless of the MHC model. In addition, those women who have used the combined MHC & CHCs evaluated the amount of the received home visits after delivery mainly as “much” (= the proportion of the “much” evaluations over 50%). The best experienced and the most received aspects of each subtheme were mainly similar in both models of the MHC. Only the aspects of the subtheme “possibility to contribute” were allocated differently in women’s and partners’ evaluations. The best aspects of each MHC service subtheme according to the model of the MHC are presented in Table 2.

**Table 2:** The best experienced and the most received aspects of each subthemes in the maternity health clinic service in relation to the clinics’ model

**MODEL OF MATERNITY HEALTH CLINIC**		
**SUBTHEME AND ASPECT OF THE MATERNITY HEALTH CLINIC SERVICE**	Separate maternity health clinic % (n)*	Combined maternity and child health clinic % (n)*
**WOMAN**		
BEST EXPERIENCE OF…		
**Group activities:**		
Regarding delivery	49.4(231)	35.3(47)
**Health promoting information:**		
Regarding nutrition	53.1(288)	53.5(83)
**Support with health problems:**		
Regarding smoking	20.2(99)	26.8(38)
**Specialist services:**		
Information regarding the services	27.0(147)	36.8(56)
MOST RECEIVED…		
**Support for the parenthood:**		
Knowledge support	70.5(391)	68.4(108)
**Parental group activity:**		
Small-groups for parents	7.6(42)	5.8(9)
**Home visit:**		
After delivery	20.0(112)	53.2(84)
**Supplement service:**		
Appointments without reservation	11.0(61)	6.5(10)
**Possibility to contribute:**		
Possibility to change PHN/midwife	-	9.7(15)
To the content of the service	7.4(41)	-
**PARTNER**		
BEST EXPERIENCE OF…		
**Group activities:**		
Regarding delivery	46.4(200)	49.6(60)
**Health promoting information:**		
Regarding nutrition	30.9(141)	32.5(40)
**Support with health problems:**		
Regarding smoking	17.2(76)	28.0(33)
**Specialist services:**		
Information regarding the services	19.6(89)	22.6(28)
MOST RECEIVED…		
**Support for the parenthood:**		
Knowledge-support	45.6(215)	42.6(55)
**Parental group activity:**		
Small-groups for parents	9.3(43)	7.6(10)
**Home visit:**		
After delivery	17.2(80)	39.9(53)
**Supplement service:**		
Appointments without reservation	5.9(27)	8.5(11)
**Possibility to contribute:**		
Possibility to change PHN/midwife	6.1(28)	-
Possibility to contribute to the content of the service	-	6.2(8)

(*) Percents and frequencies of “good” or “much” service evaluations.

(−) Not the best evaluated aspect of this subtheme in relation to the maternity health clinic’s model.

Practically all participants assessed the amount of received group activities for the fathers and home visits before delivery as “little” regardless of the MHC model. Over 85% of the participants assessed the amount of all received supplement services as “little”, and over 90% of them evaluated the amount of all parental group activities and possibilities to contribute to the MHC action as “little” regardless of the MHC model. The worst experienced and the least received aspects of each subtheme of MHC services were partly dissimilar in separate MHCs and combined MHC & CHCs. The worst aspects of each MHC service subtheme according to the model of the MHC are presented in the Table 3.

**Table 3:** The worst experienced and the least received services of each aspects in the maternity health clinic service in relation to the clinics’ model

**MODEL OF MATERNITY HEALTH CLINIC**		
**SUBTHEME AND ASPECT OF THE MATERNITY HEALTH CLINIC SERVICE**	Separate maternity health clinic % (n)*	Combined maternity and child health clinic % (n)*
**WOMAN**		
WORST EXPERIENCE OF…		
**Group activities**:		
Regarding mental health	84.5(375)	-
Regarding marital relationship	-	84.7(111)
**Health promoting information**:		
Regarding time management	85.7(455)	81.0(124)
**Support with health problems:**		
Regarding domestic violence	81.0(396)	78.9(112)
**Regarding specialist services:**		
Availability of the services	80.2(422)	75.7(115)
LEAST RECEIVED…		
**Support for the parenthood:**		
Peer support	85.0(466)	77.7(122)
**Parental group activity**:		
Groups for the fathers	98.7(549)	98.1(151)
**Home visit**:		
Before delivery	99.3(558)	96.2(151)
**Supplement service:**		
Counseling via Internet	98.4(546)	-
Group appointments	-	99.4(154)
**Possibility to contribute:**		
Possibility to change PHN/midwife	95.7(531)	-
Possibility to change or choose GP	-	92.9(144)
**PARTNER**		
WORST EXPERIENCE OF…		
**Group activities:**		
Regarding exercise	83.3(349)	-
Regarding marital relationship	-	81.9(95)
**Health promoting information:**		
Regarding time management	86.3(390)	81.0(98)
**Support with health problems:**		
Regarding overweight	-	78.0(92)
Regarding domestic violence	84.5(371)	-
**Regarding specialist services:**		
Availability of the services	80.4(364)	77.4(96)
LEAST RECEIVED.		
**Support for parenthood:**		
Peer support	85.1(399)	87.6(113)
**Parental group activity**:		
Groups for the fathers	98.3(454)	95.4(125)
**Home visit:**		
Before delivery	96.5(447)	94.8(128)
**Supplement service:**		
Counseling via Internet	-	98.5(128)
Group appointments	97.4(444)	-
**Possibility to contribute:**		
To the content of the service	96.3(442)	-
Possibility to choose or change GP	-	95.3(123)

(*) Percents and frequencies of “poor or neutral” or “little” service evaluations.

(−) Not the worst evaluated aspect of this subtheme in relation to the maternity health clinic’s model.

Statistically significant differences were found when the experiences of the participants who had used the different models of the MHCs were compared. The relations between participants’ sociodemographic background and the significant differences between the groups were examined by binary logistic regression analysis. Besides the model of the MHC also age, professional education, total income of the family, number of the children, and information on whether the pregnancy was desired or not were chosen to be the independent background variables. Most of the background variables did not explain the differences between the groups. Only the nulliparity (p < 0.001, OR 3.48, 2.43-4.97) explained the effect of the MHC model on women’s good experience with antenatal training. Differences accumulated in the combined MHC & CHCs’ favor, only group activities regarding delivery were evaluated better by women who had used the separate MHCs (Table 4).

**Table 4:** Significant differences between separate and combined maternity health clinic service in relation to good service outcomes

	**MODEL OF MATERNITY HEALTH CLINIC**			
**OUTCOME WOMAN**	**Separate maternity health clinic % (n)**	**Combined maternity and child health clinic % (n)**	**Total**	**P**
EXPERIENCES OF…				
Group activities regarding delivery				
Good	49.4(231)	35.3(47)	46.3(278)	0.004
Information regarding specialist services				
Good	27.0(147)	36.8(56)	29.2(203)	0.019
AMOUNT OF…				
Peer support				
Much	15.0(82)	22.3(35)	16.6(117)	0.030
Possibility to choose or change				
PHN/midwife				
Much	4.3(24)	9.7(15)	5.5(39)	0.010
Home visits:				
Before delivery				
Much	0.7(4)	3.8(6)	1.4(10)	0.010*
After delivery				
Much	20.0(112)	53.2(84)	27.3(196)	<0.001
Because of family or marital issues				
Much	2.3(13)	7.0(11)	3.4(24)	0.004
**PARTNER**				
EXPERIENCES OF…				
Group activities regarding smoking				
Good	22.4(94)	31.6(36)	24.4(130)	0.044
Group activities regarding intoxicants				
Good	23.2(97)	32.5(37)	25.1(134)	0.042
Support regarding intoxicant abuse problems				
Good	16.3(72)	24.6(29)	18.0(101)	0.038
Support regarding smoking problems				
Good	17.2(76)	28.0(33)	19.5(109)	0.009
Support regarding domestic violence				
Good	15.5(68)	27.1(32)	18.0(100)	0.003
AMOUNT OF…				
Home visit after delivery	17.2(80)	39.3(53)	22.2(133)	<0.001
Much				

Used statistical tests: Pearson’s Chi-Square and Fisher’s exact test (*).

## Discussion

The respondents from Southwest Finland were not entirely satisfied with the MHC services and many aspects of the service were evaluated as remarkably poor. The model of the MHC service seems to influence both women’s and partners’ experiences with several aspects of the service. Participating women were generally more satisfied with combined MHC & CHCs’ services than with those of the separate MHCs.

Participants’ poor experiences were contradictory to Viljamaa’s [26] and Perälä et al.’s [27] survey in which Finnish parents evaluated MHC and CHC work as good - measured partly by similar questions posed in the present study. One explanation for this could be that present study focused only on the content and the amount of the MHC services whereas the above mentioned researchers explored also parents’ experiences regarding the action of the public health nurse and the atmosphere of the MHCs and CHCs which were evaluated as very good in both studies. However, also critical views have been expressed by the parents. Recent Finnish studies focusing solely on maternity care services are qualitative, and they describe more censorious vision of both the women’s [30] and the men’s experiences [31]. Because of the varying study designs, the results of these studies should be compared objectively. Inconsistent findings reached by different methodological approaches uphold the existing demand for comprehensive national study focused on parents’ expectations and experiences with MHC and CHC services.

Regardless of the parents’ weak general appraisal of the MHC services in the present study, MHCs obviously have strength in supportive counselling. Especially women, but also their partners, get sufficiently supportive information from the MHCs, and professional information was conceived the most preferable form of support. This is encouraging because research shows that women expect reliable information from antenatal care providers [34,35] and adequate supportive information is related to positive experiences with maternity care [36]. On the other hand, MHC does not always seem to be the primary source of information regarding pregnancy and delivery-related topics for the expectant mothers. According to previous research, women prefer to prepare for the delivery by discussing it with friends and female relatives [37], and they actively seek information on the Internet [38]. The staff in the antenatal care should recognize and advance their role, not only as reliable information-givers, but also as professional “mirrors” for the information that parents receive from other sources, such as media and peers.

It could also be concluded that MHCs’ services are based on traditional basic elements (e.g. antenatal health check-ups and screening, health promoting counselling) and modern services, such as group appointments or diverse parental group-activities, were limited. For example, over a half of the participants in the present study reported the amount of all parental group activities as “none or very little”. Our findings regarding sparse parental group activities agree with a recent national study [27]. This is not in line with the guidelines that recommend MHCs and CHCs to arrange groups for childbearing and child rearing families [39].

The results found here show that combined MHC & CHCs might serve parents better than the separated model. Women’s higher satisfaction with combined MHC & CHCs occurred both in general and aspect-targeted assessments, whereas men reported better experiences with particular aspects of the service, such as group-based information and support with health problems. One explanation for this could be the continuing relationship with the nurse of the combined MHC & CHC that was founded during the pregnancy and will proceed until the child is at school age. Perhaps the familiarity with the nurse and the awareness of continuity of care could have made the parents evaluate the antenatal care more positively after delivery. This is supported by former national evidence indicating that parents wish to have the same nurse during pregnancy and in the CHC [26].

It also seems that the lack of continuity in MHCs and CHCs might impact the communication between the family and the care provider. According to the study of Tammentie et al. [40], the mothers whose PHN was changed after the birth of the child experienced difficulties in describing their mood and problems to an unfamiliar PHN in a CHC. In the study of Örtenstrand and Waldenström [41] Swedish women had described that their own needs, especially when there were depressive symptoms, were commonly disregarded in the CHC where they were taken care of by a different nurse than during pregnancy in the MHC. In the light of these studies, it could be speculated that parents might benefit from the continuity based service model where the same nurse will take care of them, both during pregnancy and after the birth of the child. This conclusion is supported by the multidisciplinary review of Haggerty et al. [42] which suggested that the continuity of care can improve the quality of care, regardless of the context. The relational continuity that comprises of the ongoing relationship between a patient/client and the care provider, including the shared history and future, is valued especially in primary health care settings [42]. In Finnish primary health care, this has been made possible in the combined MHC & CHCs. The results of our study could be interpreted as a manifestation of beneficial continuity in primary health care settings.

In the combined MHC & CHCs, credit should be given particularly to the home visits that provide multi-beneficial support for the families during pregnancy or postnatal period [43,44]. Women who had used the combined MHC & CHCs reported receiving home visits after delivery “much” or “very much”, nearly three times more often than women who had used the separate MHCs. Also the amount of received peer-support was reported greater by women who had used the combined MHC & CHCs. It is notable that a recent Finnish Decree on primary maternity and child health care presupposes that at least one home visit and peer support by an antenatal training group should be provided for the first-time parents [45]. The combined MHC & CHCs might be more in line with recent guidelines regarding these aspects of MHC service than the separate MHCs. However, more evidence is required to establish whether the model of the MHC is crucial for parents’ good experiences with the MHC service.

The development and optimization of the MHC services have been discussed extensively in Finland [13,15,46,47], but scarce evidence exists which model of the MHC services produces the best results in terms of parents’ experiences. The strength of this comparative study is that it has produced one of the first national reports from the perspective of both parents, and also information about parents’ poor experiences and defects in particular aspects of the MHC work. All of this could be used as a useful basis for future research and family-centered development of the MHC services.

The participation rate of the STEPS-study was low (18.3%). One main reason for this might be the challenging recruitment process; the workload of the nurses at MHCs was heavy and they did not offer the opportunity to take part in a study to all pregnant women. Moreover, the study protocol was extensive and required families’ commitment for many years which might have decreased the parents’ willingness. Despite the low participation rate of the STEPS-study, the comparison between the obstetric background characteristics of the study group and a similar non-study group from the Finnish Medical Birth Register suggests that our study effectively encompassed the parturients in the area of Turku University Hospital. Differences were found regarding women’s age, marital status, profession, and parity. However, logistic analyses showed that background variables were not notable explainers of the differences between the groups. It is known that health selection distribution caused by the low participation rate might decrease generalizability of prevalence estimates, however the associations between the studied variables could be interpreted without bias [48].

The similarity between participating and non-participating men could not be described due to the incomplete comparable background characteristics of men. The questionnaire was part of a remarkably wide research project, and questions regarding MHC services were included in a multidisciplinary questionnaire containing several thematic parts. These details could account for the limitations of the study.

## Conclusions

These results suggest that the organizational model of the MHC might have an influence on parents’ experiences. The continuity of care in the combined MHC & CHCs seems to increase parents’ satisfaction with the specific aspects of the care. Moreover, the model of the combined MHC & CHCs provides more home visits and peer support than that of the separate MHC.

The experiences and wishes of the parents should be taken into account when optimizing maternity care services. However, the health and the well-being of the mother, baby, and the whole family are the principal objectives of maternity care; hence the comparison of the different maternity care models should include rigorous evaluation of the maternal and perinatal health outcomes. Accordingly, our future research will be exploring the relationship between MHC’s organizational model and the health outcomes of the mother and the baby.

## Abbreviations

MHC: Maternity health clinic; CHC: Child health clinic; MHC & CHC: Combined maternity and child health clinic; PHN: Public health nurse; GP: General practitioner.

## Competing interests

The authors declare that they have no competing interests.

## Authors’ contributions

MT, PA and PR were involved in the study concept, design, and the acquisition of data. AK and MT performed the statistical analyses. MT drafted the manuscript; all authors were involved in the review and approval of the final manuscript. All authors were involved with the interpretation of the data and have read and approved the final manuscript.

## Pre-publication history

The pre-publication history for this paper can be accessed here:

http://www.biomedcentral.com/1471-2393/12/96/prepub
